# 

**Published:** 1895-06-29

**Authors:** 


**Notices of Births, Deaths, and Marriages** are inserted in this column at a charge of 2s. 6d. for an announcement not exceeding four lines.

**Notice to Advertisers and Subscribers.**

All Advertisements, Orders for Copies of The Hospital and Business Communications should be addressed to **The Manager, NOT TO THE EDITOR**, The Hospital, 428, Strand, London, W.C.

The Cost of Subscription, post free, is as follows:—Per week, 2½d.; per quarter, 2s. 8d.; per half-year, 5s. 3d.; per annum, 10s. 6d. Foreign:—Per Annum, 15s. 2d.; payable, in all cases, in advance.

### NOTICE TO CORRESPONDENTS.

All MS., Letters, Books for Review, and other matters intended for the Editor should be addressed The Editor, The Lodge, Porchester Square, London, W.

The Editor cannot undertake to return rejected MS., even when accompanied by stamped directed envelope.

**"Burdett's Hospital Annual," 1895,**
Now Ready.

Sixth year of publication. 950 pp. Scarlet cloth, gilt lettered. Price 5s. A most complete guide to British, Colonial, Indian, and American Hospitals, Dispensaries, Nursing and Convalescent Institutions, and Asylums. A few copies of the "Annual" for 1894 may still be obtained on application to the Publishers, The Scientific Press (Limited), 428, Strand, London, W.C.

**NOTICE.—Vol. XVII. of "The Hospital" (October, 1894, to April, 1895), in handsome cloth binding, is now on sale at the Office of this Journal. Price 6s. Cases for binding the Numbers contained in this Volume, 1s. 6d. Index to Vol. XVII., 2½d., post free.**

### SCRAPS AND GLEANINGS.

The munificent legacy of £36,236 has been left to the London Hospital by Mr. W. A. Gursden.

A meeting was held at 4, St. James's Square in support of the Children's Holiday Fund, at which Lord Cowper presided.

Mr. J. B. Robinson, of Dudley House, Park Lane, has sent £1,000 to the Lord Mayor as a donation to the Hospital Sunday Fund.

On the recommendation of the Indian Government the Queen has approved the bestowal of a good service medal on Surgeon Major-General McVittie.

A demonstration and parade is arranged to be held on July 7th at Parkstone in favour of the funds of the Parkstone and Newtown Hospitals.

Princess Christian will present certificates and medallions on July 12th to the pupils of the Norwood centre of the St. John Ambulance Association.

The Duchess of Albany has just visited the Royal Hospital for Children and Women, Waterloo Road, and left a choice collection of flowers for the patients.

Towards the proposal for a new Emergency Hospital for Boston, a lady resident of that city has offered to contribute 250,000 dollars. This will be as a memorial to her husband.

A new room for the eye department of Addenbrooke's Hospital is to be erected forthwith, the expenses of which will be paid out of the balance of the Sir George Humphrey's Donation Fund.

M. Th. Fr. Ritter has given to the Faculty of Medicine at Nancy a sum of 18,000 francs, the interest of which is to be employed in the formation of a biennial prize to be named the Ritter Prize.

An "At Home" was also held at 83, Lexham Gardens, by Lady Borthwick, when a distinguished company assembled to support the interests of the Church of England Waifs and Strays Society.

In response to the appeal at the festival dinner in aid of the funds of the Royal Maternity Charity held recently donations amounting to about £800 were announced, including one of £50 from the Queen.

Miss Peabody has left the Catholic University of Washington the residue of her estate to endow scholarships in the chemical and physical sciences. These will probably consist of four of the value of about £100 each.

At Tonquin the Hanoi Hospital has been officially inaugurated. The sanitary and all internal arrangements have had the greatest consideration paid to them. The hospital will afford accommodation for 300 beds.

A correspondent states that Mr. J. G. Bryant, the collector at the Metropolitan Hospital, Kingsland Road, has, by personal application, obtained, in addition to subscriptions, £550 in donations during the past six weeks.

We congratulate the people of Woolwich on the new recreation ground which has been granted them through the generosity of Mr. Passmore Edwards and the untiring zeal of the Metropolitan Public Gardens Association.

During 1892 the total number of vaccinations and re-vaccinations in Germany amounted to 2,420,161, out of which 18,188 were done with humanized lymph. The following year there were 25 stations in Germany for the supply of animal lymph.

A special service was held last Sunday at St. Margaret's, Westminster, on behalf of the Royal College for the Blind at Norwood, the blind from the college rendering the musical part of the service. The offertory was devoted to the funds of the institution.

Measures are now being definitely taken at Reigate to provide the district with adequate accommodation for infectious diseases. This accommodation, indispensable for a population approaching 30,000, ought, of course, to have been supplied long ago.

A sale of work, consisting of goods left over from the bazaar promoted last autumn in favour of the funds of the Fleetwood Cottage Hospital, has just been held in the town. Over £100, including donations, were received at the end of the first day's sale.

The Smallwood Hospital at Redditch has now been formally opened by Lady Windsor. This is the outcome of one of the many charitable bequests of the late Mr. Edwin Smallwood, supplemented subsequently by larger sums from Mr. William Smallwood, Alcaster.

The Czar is about to reopen the institute for women at St. Petersburg. This establishment was closed by order of the Government several years ago. It will be permissible for former students who have attained their degree to undertake the work of medical practitioners.

Sir J. Pease, M.P., presided at the annual meeting of the Society for the Suppression of the Opium Trade at the Westminster Town Hall recently, and said that, so far from the report of the Commission being a serious blow to the anti-opium agitation, it was one of the most important anti-opium documents issued.

In the tabular statement of a year's work at the metropolitan hospitals, published in our special supplement of the 15th inst., the income figures of University College Hospital in the columns headed "Proprietary" and "Patients' Payments," were accidentally transposed. The correct figures are, proprietary income, £5,041; patients' payments, £28.

Mr. H. M Stanley has made a curious proposal in aid of the London hospitals. His suggestion is that the governors of the institution should select a load of human specimens from the children's wards and parade them in a waggonette through the West-end of London. This hospital circus, as it has been remarked, would certainly be a novelty in the streets of our Metropolis!

A German contemporary gives us curious facts respecting the condition of bath water, after a bath has been taken, with regard to microbic deposit. The correspondent states that water thus examined he found to contain eight hundred and fifty million micro-organisms. No less than one hundred and eighty millions he estimates further will be found after bathing one foot of a human being.

### The Student of Medicine.

### APPOINTMENTS.

T. D. Acland, M.A., M.D.Oxon, F.R.C.P.Lond., appointed Physician to the Hospital for Consumption, Brompton. Dr. Ambler, appointed Resident Medical Officer to the Birkenhead Workhouse. Miss Louise Appel, M.B.Lond., appointed Assistant to the Anæsthetist at the Royal Free Hospital. C. Y. Biss, B.A.Cantab., M.D., F.R.C.P.Lond., appointed Physician to the Hospital for Consumption, Brompton. A. Bowhay, D.P.H. Cantab., L.R.C.P.Lond., M.R.C.S.Eng., appointed Medical Officer of Health to the Calstock Rural District Council. Dr. P. Butler, appointed Medical Officer for the Chiddingfold District of the Hambledon Union. T. H. Butler, L.R.C.P., M.R.C.S., appointed Resident Medical Officer (House Physician) to the Royal Free Hospital. C. D. Christmas, L.R.C.P.Lond., M.R.C.S. Eng., appointed Medical Officer for the Streatham District of the Clapham Union. J. Davidson, M.B.Lond., M.R.C.S., appointed Medical Officer to the Uxbridge Joint Hospital. H. B. Gardner, M.R.C.S.Eng., L.R.C.P.Lond., appointed Assistant Anæsthetist to the Charing Cross Hospital. H. R. Gardener, M.B., M.S. Aberd., appointed Second Assistant Medical Officer to the Waterloo Road Workhouse of the Bethnal Green Union. T. H. Gillam, L.R.C.P.Lond., M.R.C.S., appointed Medical Officer for the Sapey Pitchard District of the Martley Union. Miss K. Marion Hunter, L.S.A., appointed Assistant to the Anæsthetist at the Royal Free Hospital. B. S. Lockwood, L.R.C.P., L.R.C.S.Edin., appointed Assistant Medical Officer to the Leeds Union Workhouse. Mr. McKeith, appointed Medical Officer for the Ilfracombe District of the Barnstaple Union. A. J. Martin, M.B., M.R.C.S., appointed Medical Officer for the Bloxwich District of the Walsall Union. J. T. Martin, L.R.C.P., L.R.C.S.I., appointed Medical Officer of the Catholic training-ship *Clarence*. C. J. O'Connor, L.R.C.P.I., F.R.C.S.I., appointed Dispensary Medical Officer for the Celbridge District. S. Riseley, M.D.Edin., appointed House Surgeon to the Rotherham Hospital and Dispensary. C. F. Rudd, M.R.C.S.Eng., L.S.A., appointed Medical Officer for the Stalham District of the Smallburgh Union. G. Schorstein, M.A.Oxon., M.B., M.R.C.P. Lond., appointed Assistant Physician to the Hospital for Consumption, Brompton. H. B. Seddon, L.R.C.P.Lond., M.R.C.S. Eng., appointed Honorary Out-patient Surgeon to the Newport and Monmouthshire Infirmary. T. W. Smith, L.R.C.P., M.R.C.S., appointed Resident Medical Officer (House Surgeon) to the Royal Free Hospital. G. S. Stansfield, L.R.C.P., M.R.C.S., appointed Non-resident Medical Officer to the Birkenhead Workhouse. R. E. Thompson, M.D.Cantab., F.R.C.P.Lond., appointed Consulting Physician to the Hospital for Consumption, Brompton. A. L. Webb, M.R.C.S., M.R.C.P., appointed Resident Medical Officer to the National Hospital for Diseases of the Heart and Paralysis, Soho Square. H. W. Webber, M.D., M.S.Lond., appointed a Divisional Surgeon to the Herts County Police. F. J. Wethered, M.D.Lond., M.R.C.P.Lond., appointed Assistant Physician to the Hospital for Consumption, Brompton. E. Wilcox, M.D., appointed Medical Officer for the Ashburton District of the Newton Abbott Union. T. Williams, M.A.Oxon., M.D., F.R.C.P.Lond., appointed Consulting Physician to the Hospital for Consumption, Brompton.

### VACANCIES.

The following vacancies are announced this week, the amount of salary in each case being given after the name of the institution:

### Resident Medical Officers.

Cork District Lunatic Asylum.—Two Assistant Medical Officers, unmarried, and not more than 30 years of age, doubly qualified. Salaries, £100 per annum, with furnished apartments, rations, washing, fuel, light, and attendance. Applications to the Resident Medical Superintendent by June 29th.

Sheffield General Dispensary.—House Surgeon and Senior Assistant House Surgeon, doubly qualified. Salary for the former, £120 per annum, with a prospective advance of £10 per year for the second and third years; and for the latter £80 per annum, with board, lodging, and washing. Applications to the "Medical Staff of the Sheffield General Infirmary, to the care of the Secretary," by July 13th.

Victoria Hospital for Children, Queen's Road, Chelsea, S.W.—House Physician to the In-patients. Honorarium, £50 per annum, with board and lodging in the hospital. Applications to the Secretary by June 29th.

### Non-Resident Medical Officers.

London Hospital, Whitechapel, E.—Medical Electrician. Applications to the House Governor by June 29th.

London Hospital Medical College, Mile End.—Senior Demonstrator of Anatomy. Salary payable by a percentage of fees. Applications to Munro Scott, Warden, by July 8th.

St. Marylebone General Dispensary, 77, Welbeck Street, Cavendish Square.—Honorary Dental Surgeon. Applications to the Secretary by July 1st.

University of Aberdeen.—Eleven Examiners. Appointment for one year. The Examiners in Medicine and Surgery will receive grants of £50, and the other nine Examiners £40. Applications to Robert Walker, Secretary to the University Court, July 3rd.

